# Safety and efficacy of Levorag emulgel in the treatment of anal fissures using a validated scoring system

**DOI:** 10.3389/fsurg.2023.1145170

**Published:** 2023-03-22

**Authors:** G Tomasicchio, A Dezi, A Picciariello, D. F Altomare, C Giove, G Martines, M De Fazio, M Rinaldi

**Affiliations:** Department of Precision of Regenerative Medicine and Ionian Area, University “Aldo Moro” of Bari, Italy

**Keywords:** anal fissure, topical treatment, anal pain, scoring system, healing

## Abstract

**Introduction:**

Anal fissure is one of the most common anal disease characterized by intense anal pain, and deterioration of patients quality of life. Treatment is mainly based on the topical administration of calcium antagonist or nitric oxide ointments, and in cases refractory to medical treatment patients can undergo surgery. This study aims to assess the efficacy and safety of Levorag emulgel in the treatment of acute and chronic fissures using of a validated scoring system.

**Material and Methods:**

A prospective observational study was carried out on patients with anal fissures between February and May 2022. The efficacy of the treatment was evaluated using the REALISE score, a new validated scoring system that rates VAS for pain, NSAID use, pain duration, bleeding, and quality of life (QoL), recorded after 10, 20 and 30 days from the beginning of treatment.

**Results:**

Forty patients (median age 46 years, IQR 29–57, 70% women) with acute (22, 55%) or chronic (18, 45%) anal fissures entered the study. The median anal pain score according to the VAS scale decreased significantly from 7 (IQR 4.7–8) at baseline to 1 (IQR 0–3.2, *p* = 0.05) after 20 days. At the 30-day proctological examination, 22 patients (61%) were pain free (median VAS of 0, IQR 0–1.2, *p* < 0.05). Pain duration after defecation measured according to the REALISE score, showed a significant decrease after 10 days, from a median value of 2 (IQR 1–4) to 1 (IQR 1–1.2) (*p* < 0.005). The median value of the REALISE score decreased significantly, from 15 (IQR 11–19.25) at first proctological evaluation to 4 (IQR 4–6, *p* = 0.139) after 30 days of treatment. At day 30, complete fissure healing was achieved in 30 patients (80%). The healing rate was 82% and 78% in patients with acute and chronic anal fissures, respectively.

**Conclusion:**

The use of Levorag® Emulgel may represent a safe and effective non-invasive first line treatment in patients affected by acute or chronic anal fissure.

## Introduction

Anal fissure (AF) is one of the most common anal disease characterized by intense and prolonged anal pain after defecation, bleeding, and considerable deterioration of patient's quality of life (1). AF is often caused by the passage of hard stools or prolonged diarrhea (2). Spontaneous healing rarely occur because the reactive spastic contraction of the internal anal sphincter, decreases blood flow to the affected area (1–4). In fact, treatment strategies aim to reduce this uncontrolled spasm, using topical creams based on calcium antagonists (such as diltiazem) or nitric oxide donors (such as glycerin trinitrate) (5, 6) or by injections of botulinum toxin A (7) allowing restoration of blood flow to the anoderm (8) and to facilitate evacuation by using stool softeners.

When conservative treatments fail, surgical options including internal anal sphincterotomy, anal advancement flap, and anal stretch/dilation may be considered (9, 10).

None of these medical treatments are completely free from side effects such as headaches, migraines, and pruritus ani, while surgical approach can cause bleeding, abscesses, fistulas, and considerable risk of fecal incontinence (11–13).

This study aims to evaluate the safety and effectiveness of Levorag® Emulgel, a topical ointment which favors pain relief, microperfusion of the anoderm and the reepithelization process(11, 14), in the treatment of acute and chronic anal fissures assessed by a new validated scoring system.

## Material and methods

A prospective observational study was carried out in a tertiary proctology unit on patients with anal fissures between February and May 2022. After receiving approval from the local ethics committee, patients of both sexes aged 18 to 85 years with acute or chronic anal fissures were enrolled. Exclusion criteria included previous medical or surgical treatment, perianal Crohn's disease, previous anorectal operations, prolapsed hemorrhoids, rectal prolapse, functional disorders of the defecation and continence, and pregnancy. Acute fissures were defined as recent (within 6 weeks) ulcerations of the anoderm, while chronic anal fissures persisted for more than 6 weeks, with sentinel tags and/or induration of the lateral margins of the fissure and/or exposure of the internal anal sphincter. Demographic data and detailed clinical histories were recorded, including information about anal pain intensity and duration, bowel habits, and stool consistency (using the Bristol stool scale) (15) before treatment. Patients were examined in the Sims position. The anal verge was inspected to confirm the presence of the anal fissure and to determine its location. When tolerated, digitorectal examination (DRE) to evaluate the anal sphincter tone and anoscopy were performed. Eligible patients received three boxes of Levorag® Emulgel (THD, SpA, Coreggio, RE, Italy), a topical ointment containing a Hibiscus plant extract called myoxinol with a botox-like effect, and carboxymethyl glucan, a yeast polysaccharide with immune-stimulating properties, for use in the conservative treatment of anal fissures. Each box contained twenty 3.5 ml single-dose tubes. Patients were instructed to apply the ointment twice a day (every 12 h) for 30 days using the tip of their finger. Stool softeners were administered in case of constipation. The efficacy of the treatment was evaluated using the REALISE score, a new validated scoring system that rates VAS for pain, NSAID use, pain duration, bleeding, and quality of life (QoL) (16). The score was calculated during the first clinical evaluation, at day 10 and 20 by a telephone interview (17, 18), and at day 30 by an outpatient evaluation. Side effects were recorded. The degree of re-epithelization (healing) was evaluated and scored as follows: 0 = anal fissure still present, 1 = superficial fissure, 2 = partial re-epithelization, 3 = complete re-epithelization. Patient satisfaction was rated on a scale of 0 (failure) to 5 (excellent).

## Statistical analysis

Continuous parameters were reported as median and interquartile ranges. Categorical variables were recorded as numbers and percentages. Comparisons of categorical variables were performed by the Chi-square and Fisher's Exact test, where appropriate. Comparisons between groups were made by the Mann-Whitney U test. A *p* value < 0.05 was considered statistically significant. Statistical analysis was carried out using RStudio (R version 4.0.3 (2020-10-10) Copyright (C) 2020 The R Foundation for Statistical Computing).

## Results

Forty patients (median age 46 years, IQR 29–57, 70% women) with acute (22, 55%) or chronic (18, 45%) anal fissures entered the study after giving an oral informed consent.

**Figure 1:**
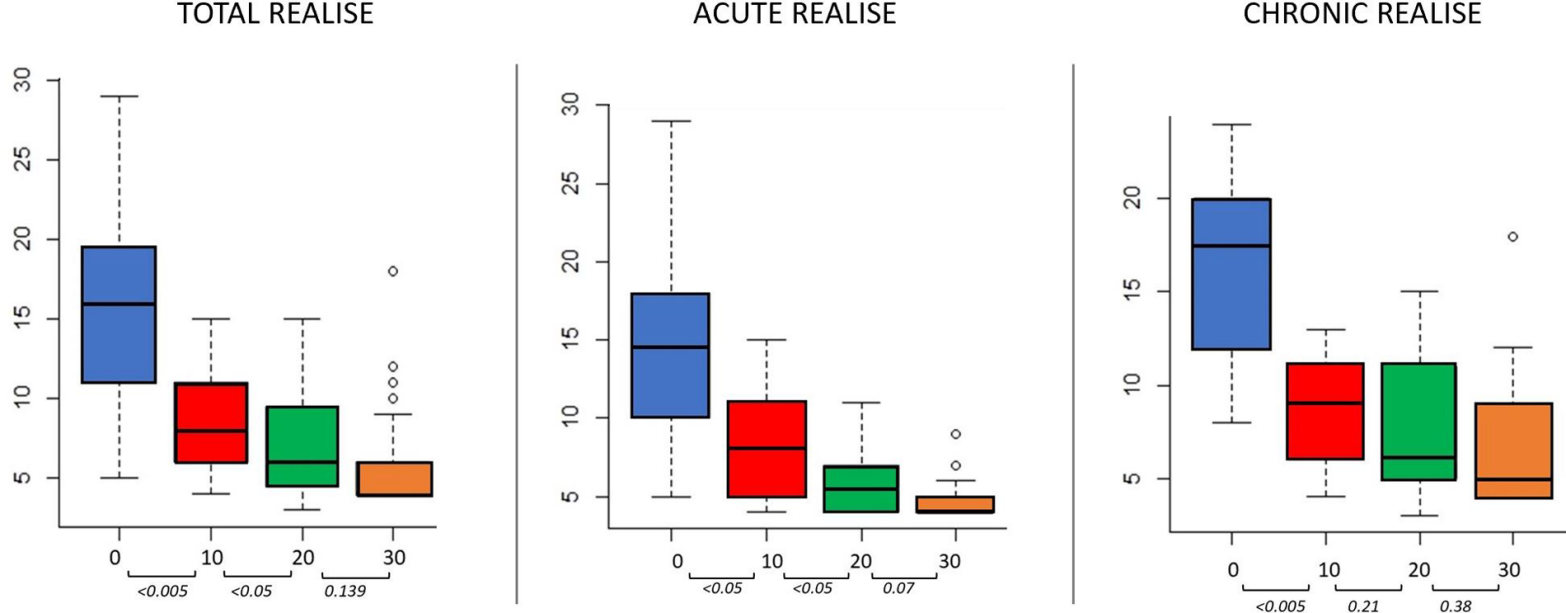
REALISE score at the baseline, 10–20 and 30 days from the start of treatment.

Thirty-six patients (12 males, median age 52 years, IQR 28–57, and 24 females, median age 45 years, IQR 29–56) completed both telephone interviews and a proctological evaluation at day 30. Four out of forty patients (10%) who did not complete the follow-up, underwent internal anal sphincterotomy (1 patient) and calcium antagonist-based ointments 10 days after the first consultation (3 patients). Previous pregnancies were reported by 14 women (58%) (Table 1). Four patients (11%) had an anterior anal fissure. digital rectal examination (DRE) was not tolerated at the first consultation by 10 patients (28%). The median anal pain score according to the visual analogue scale (VAS) was 7 (IQR 4.7–8) at baseline, and decreased to 3 (IQR 1–5, *p* < 0.005) 10 days later and to 1 (IQR 0–3.2, *p* = 0.05) after 20 days. At the 30-day proctological examination, 22 patients (61%) were pain free (median VAS of 0, IQR 0–1.2, *p* < 0.05). Pain duration after defecation measured according to the REALISE score, showed a significant decrease after 10 days, from a median value of 2 (IQR 1–4) to 1 (IQR 1–1.2) (*p* < 0.005). This score did not change after 20 and 30 days (median value 1, IQR 1–1, *p* = 0.23). With regard to bowel habits at the first evaluation, 8 patients (22%) had stool type 2 according to the Bristol stool scale, while 24 (67%) and 4 (11%) patients had type 3 and 6, respectively. The median value of the REALISE score at the first clinical evaluation was 15 (IQR 11–19.25) with a significant reduction at 10 days to 8 (IQR 6–11, *p* < 0.005). At 20 days, a median value of 6 (IQR 4.75–6.25, *p* < 0.05) was recorded. Between 20 and 30 days after the onset of treatment, the score decreased to 4 (IQR 4–6, *p* = 0.139) (Figure 1). At day 30, complete fissure healing was achieved in 30 patients (80%). One patient still had a chronic anal fissure at 30 days follow-up. Partial healing with complete symptoms remission was recorded in 5 patients. The healing rate was 82% and 78% in patients with acute and chronic anal fissures, respectively. At the last consultation, satisfaction was scored as 5 (extremely satisfied) by 25 patients, 4 (satisfied) by 7 patients, 3 (moderately satisfied) by 2 patients, 2 (not satisfied) by 1 patient and 1 (completely not satisfied) by the last one. Eight patients complained of pruritus ani during the first examination, with complete relief 20 days after. No other adverse events were recorded. All domains of the REALISE score are reported in Table 2.

**Table 1:** Demographic characteristics of patients included in the study.

	*n* = 36
**Gender**
- M	12 (33%)
- F	24 (67%)
**Pregnancy**
- 0	10 42%
- 1	25%
- 2	7 29%
- 3	1 4%
**Type of fissure**
- Acute	22 (61%)
- Chronic	14 (39%)

**Table 2:** RALISE domains at the baseline, 10-20 and 30 days from the start of treatment.

	First evaluation	10 days	20 days	30 days
VAS	7 (4.75–8)	3 (1–5)	1 (0–3.25)	0 (0–1.25)
		*p* < 0.005	*p* = 0.05	*p* < 0.05
Pain duration	2 (1–4)	1 (1–1.25)	1 (1–1)	1 (1–1)
		*p* < 0.005	*p* = 0.239	*p* = 0.239
**NSAID use**
- Never	25 (69.5%)	33 (92%)	34 (94%)	34 (94%)
- Rarely	6 (16.5%)	3 (8%)	1 (3%)	1 (3%)
- Sometimes	2 (5.5%)	0	1 (3%)	0 (3%)
- Often	2 (5.5%)	0	0	1
- Alwalys	1 (3%)	0	0	0
		*p* < 0.005	*p* = 0.05	*p* < 0.05
**Bleeding**
- Never	14 (39%)	28 (78%)	32 (89%)	31 (86%)
- Rarely	11 (31%)	8 (22%)	4 (11%)	4 (11%)
- Sometimes	6 (16.5%)	0	0	0
- Often	3 (8%)	0	0	1 (3%)
- Alwalys	2 (5.5%)	0	0	0
		*p* < 0.005	*p* = 0.21	*p* = 0.54
**QoL**
- No impact	4 (11%)	10 (28%)	21 (58%)	26 (72.5%)
- Slightly	5 (14.5%)	13 (36%)	11 (31%)	6 (16.5%)
- Moderately	8 (22%)	12 (33%)	4 (11%)	3 (8%)
- Considerably	16 (44.5%)	1 (3%)	0	1 (3%)
- Severely	3 (8%)	0	0	0
		*p* < 0.005	*p* < 0.005	*p* = 0.64
REALISE	15 (11–19.25)	8 (6–11)	6 (4.75–9.25)	4 (4–6)
		*p* < 0.005	*p* < 0.05	*p* = 0.139

## Discussion

Non-operative management is the first-line approach for treating anal fissures, as it can provide relief the associated anal spasm with pain decreases and healing of the fissure in about 60 to 80% of the cases (19).

A recent survey among gastrointestinal surgeons in the Netherlands reported that initial treatment consists of conservative measures including administration of fibers/laxatives and topical ointments (20).

In the past few decades, several topical ointments have been proposed as non-invasive treatments for anal fissures. Currently, the Association of Coloproctologists of Great Britain and Ireland recommends diltiazem as first-line treatment for chronic anal fissures (21) with a healing rate of 52.3% after 8 weeks, because of its lower side effect (22), despite the use of topical nitrate has showed a higher healing rate (63.6%). However, in a recent randomized clinical trial on patients affected by chronic anal fissures, glyceryl trinitrate ointment resulted less effective than tocopherol acetate in the reduction of anal pain and in term of healing and recurrence rate 16 weeks after finishing the treatment (23).

Botulinum toxin can also be used as conservative treatment being more effective than nitrates and calcium channel blockers even if but the local injection is painful and its effect is temporary (7).

In this study, the administration of Levorag® Emulgel, a topical ointment with natural anti-inflammatory agents, botox-like effect, and immune-stimulating properties, resulted in complete healing in 80% of patients with acute or chronic anal fissures. These results are in agreement with data published by Digennaro et al. (11) who reported an efficacy of 89.4% in acute and 62.8% in chronic anal fissures in their prospective multicenter observational trial on 265 patients.

Nordholm-Cartensen et al. (24) reported a healing rate of 52% in a randomized clinical trial of patients with chronic anal fissures, although the number of patients in the Levorag group (26 patients) was smaller than the estimated sample size and the study's power was only 70%. Furthermore, the RCT did not provide a clear definition of “fissure healing.”

A preliminary study by Giordano et al. (25) on the use of Levorag in patients with chronic anal fissures, reported a healing rate of 84% with over 85% of bleeding control after 40 days of treatment. Our study confirms these results, but the Levorag ointment was administered for only 30 days. In addition, our data showed a significant reduction of pain after 10 days of treatment (7 vs. 3 on the median VAS), with a slow but progressive pain reduction in the follow-up. Furthermore, the introduction of a validated score to assess the severity of the fissure, makes the evaluation of our results more objective and measurable.

In our analysis, the healing rate was higher than other medical treatments using topical ointments, however the small sample size and the inclusion of acute anal fissures may limit the reliability of the study. Other limitations of this study are the lack of a control group and the use of only the Bristol scale to evaluate bowel habit.

In conclusion the use of Levorag® Emulgel may represent a safe and effective non-invasive first line treatment in patients affected by acute or chronic anal fissure. Multicenter prospective randomized studies with long-term follow-up are expected to confirm our results.

## Data Availability

The raw data supporting the conclusions of this article will be made available by the authors, without undue reservation.
